# Allogeneic stem cell transplant recipients surviving at least 2 years without relapse: outcome and risk factors

**DOI:** 10.1002/jha2.842

**Published:** 2024-02-05

**Authors:** B. Linder Grønvold, Maryan Mohamed Ali, Tor Å Myklebust, Andrea Lenartova, Mats Remberger, Ingerid Weum Abrahamsen, Geir Erland Tjønnfjord, Anders Eivind Myhre, Yngvar Fløisand, Tobias Gedde‐Dahl

**Affiliations:** ^1^ Department of Haematology Oslo University Hospital Oslo Norway; ^2^ Institute of Clinical Medicine University of Oslo Oslo Norway; ^3^ Department of Registration Cancer Registry Norway Oslo Norway; ^4^ Department of Research and Innovation Møre and Romsdal Hospital Trust Ålesund Norway; ^5^ Department of Medical Sciences Uppsala University and KFUE Uppsala University Hospital Uppsala Sweden; ^6^ Center for Cancer Cell Reprogramming Institute of Clinical Medicine University of Oslo Oslo Norway

**Keywords:** aGvHD, Allo‐HSCT, cGvHD, GvHD‐prophylaxis, long‐term follow‐up

## Abstract

Outcomes of 2‐year survivours undergoing allo‐haematopoietic stem cell transplantation at Oslo University Hospital were retrospectively assessed with the objectives of identification of risk factors for late death as possible means for precautionary measures and interventions to improve long‐term survival.

421 patients with haematological malignancy, transplanted between 2005 and 2019, alive and free of disease after 2 years were included with data reported from The OUS‐HSCT registry. Median follow‐up was 6.2 years (2.016.1), and 232 patients (55%) were observed for minimum 5 years.

The probability of being alive 5 and 10 years after HSCT was 86% and 76%. Primary risk factors for late death included initial diagnosis of age ≥ 60 years, chronic lymphocytic leukaemia (CLL), previous blood stream‐ or invasive fungal infection (BSI, IFI), and chronic graft‐versus‐host disease (cGVHD). Transplant‐related mortality (TRM) and relapse at 5 years were 9.0% and 7.7%, respectively. Two factors were associated with the latter: cytomegalovirus (CMV) seronegative donor and CLL. Compared with the age‐ and gender‐matched Norwegian general population, life expectancy was lower for each disease, except for CML.

The prospect for the long‐term survival is good for 2‐year survivors of the allogeneic hematopoietic stem cell transplantation. However, life expectancy remains inferior to the age‐ and gender‐matched general population. Optimising prophylaxis and treatment for chronic GVHD, BSI and IFI are needed along with the improved adherence to guidelines for early detection of secondary malignancies. Measures to improve immune reconstitution, possibly the microbiota, and the use of CMV seropositive donors regardless of recipient sero‐status may be warranted and should be addressed in further studies.

## Graphical abstract

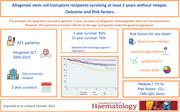

## 1 BACKGROUND

Outcome after allogeneic haematopoietic stem cell transplantation (HSCT) has improved in recent years [1] even though patients are older and alternative donors are used more frequently [2]. Reasons for this are various, including use of less toxic conditioning protocols, advancement in human leukocyte antigen (HLA)‐typing and extended donor availability, increased knowledge on prophylaxis and treatment of graft versus host disease (GVHD), better supportive care, testing for minimal residual disease (MRD), use of new prognostic models, and measures to prevent relapse [3, 4].

Most deaths’ post transplantation occurs during the first 2 years [5, 6]. In patients who survive longer, the leading causes of long‐term mortality are chronic GVHD, relapse of malignant disease, infections and secondary malignancies [7]. Compared to the general population, survivors beyond 2‐ and even 5 years still have lower life expectancy and two‐ to nine‐fold increased mortality ranging in studies [8].

In the largest retrospective study to date, the estimated 10‐year survival was 85% in 2‐year survivors, based on records from the Centre of International Blood and Marrow Transplant Research (CIBMTR) [9].

In this study, we evaluated the outcome of patients who were alive without relapse 2 years after HSCT, and compared survival of these patients with an age‐ and gender‐matched Norwegian population. Our main objective was identification of risk factors for late death as possible means for precautionary measures and interventions to improve long‐term survival.

## 2 PATIENTS AND METHODS

### 2.1 Patients

Adult (≥18 years) patients with malignant disorder who underwent allo‐HSCT at OUS from 2005 to 2019 and were alive without relapse 2 years post‐transplant were eligible for the study. OUS is a national referral centre conducting the vast majority of allo‐HSCT in Norway. Patient characteristics are displayed in Table 1. The study was approved by the Regional Committee for Medical and Health Research Ethics of South‐East Norway. The procedures were in accordance with the Helsinki Declaration.

**TABLE 1:** Characteristics of patients alive without relapse 2 years after HSCT.

Factor	*N*, median (range)
**Age**	50 (18–72)
**Sex (M/F)**	252/169
**Diagnoses**:	
AML	216 (50)
ALL	32 (7)
CML	17 (4)
Lymphoma	68 (16)
CLL	12 (3)
MDS/MPS	76 (18)
**Stage**:	
Early/late	198/223
**Donor**:	
MRD	109 (26)
MUD	253 (60)
MM URD	48 (11)
Haplo	11 (3)
**Donor age**	31 (15–72)
**FtoM**	69 (16)
**Rec CMV sero +**	308 (73)
**Donor CMV sero +**	189 (46)
**Source**:	
BM/PBSC	110/311
**CD34 cell‐dose** (×10^6^/kg)	6.5 (0.6–21.4)
**Conditioning**:	
**MAC/RIC**	228/193
**ATG**	81 (19)
**GVHD proph**:	
CsA/Tac	10 (2)
CsA+MTX	323 (77)
CsA+MMF	6 (1)
CsA+Sirolimus	65 (15)
PTCy	17 (4)
**Previous acute GVHD**:	
**0–I**	275 (65)
**II**	106 (25)
**III–IV**	40 (10)
**Chronic GVHD**:	
No	143 (34)
Limited	141 (33)
Extensive	137 (33)
**Previous infections**:	
BSI	253 (60)
IFI	50 (12)
CMV infection	163 (39)

Abbreviations: ALL, acute lymphoid leukaemia; AML, acute myeloid leukaemia; ATG, anti‐thymocyte globuline; BM, bone marrow; BSI, blood stream infection; CLL, chronic lymphoid leukaemia; CML, chronic myeloid leukaemia; CMV, cytomegalovirus; CsA, cyclosporine; early stage, CR1/CP1; FtoM, female‐donor‐to‐male recipient; haplo, haploidentical donor; HSCT, haematopoietic stem cell transplantation; IFI, invasive fungal infection; late stage, beyond CR1/CP1; MAC, myeloablative conditioning; MDS, myelodysplastic syndrome; MMF, mycofenolate mofetil; MMURD, mismatched unrelated donor; MPN, myeloproliferative neoplasm; MRD, matched related donor; MUD, 10/10 matched unrelated donor; PBSC, peripheral blood stem cells; PTCy, port‐transplant cyclophosphamide; RIC, reduced intensity conditioning; Tac, tacrolimus.

### 2.2 HLA typing

All patients and donors were typed using polymerase chain reaction (PCR)‐SSP high‐resolution typing for both HLA class I and II alleles.

### 2.3 Donors

An HLA‐identical sibling donor was used in 109 (26%) cases, a 10/10 matched unrelated donor (MUD) in 253 (60%) and a 9/10 matched unrelated donor (MMURD) in 48 (11%) cases. In 11 (3%) cases, a haploidentical‐related donor was used. Median donor age was 31 (15–74) years. A female donor‐to‐male recipient situation was used in 69 (16%) cases.

### 2.4 Conditioning regimen and GVHD prophylaxis

Reduced intensity conditioning (RIC) was given to 193 patients and consisted of fludarabine 120–150 mg/m^2^ in combination with (a) cyclophosphamide (Cy) 2400 mg/m^2^ (*n* = 73), (b) treosulfan 42 g/m^2^ (*n* = 42) or (c) 8 mg/kg busulfan (Bu) orally (*n* = 72), (d) 2 Gy total body irradiation (TBI) (*n* = 3). Three patients received other combinations. Myeloablative conditioning (MAC) (*n* = 228) consisted of Cy 120 mg/kg in combination with (a) TBI 13 Gy (*n* = 16) or (b) either steady‐state concentration tailored p.o. Bu 16 mg/kg or i.v. Bu 3.2 mg/kg (*n* = 191). Twelve patients received Bu + thiotepa + fludarabine. Eight patients received fludarabine and Bu 16 mg/kg.

Prophylaxis against GVHD consisted of cyclosporine A (CsA) or tacrolimus alone (*n* = 10) or in combination with methotrexate (MTX, *n* = 323), mycophenolate mofetil (MMF, *n* = 6) or sirolimus (*n* = 65). Seventeen patients transplanted with an HLA‐haploid family donor received CsA+MMF and post‐transplant cyclophosphamide. During the first month, blood CsA trough levels were kept at 200–300 ng/mL, depending on the transplant protocol. In the absence of GvHD, CsA was tapered with the aim of discontinuation after 4–6 months. Anti‐thymocyte globulin (Thymoglobulin, ATG), 4 mg/kg in 10/10 MUDs‐ or 6 mg/kg in 9/10 MUD transplants, was given to 81 (19%) patients.

### 2.5 Stem cell source

Three hundred and twelve patients (74%) received peripheral blood stem cells (PBSC) and 110 received bone marrow (BM).

### 2.6 Statistics

Overall survival (OS) and relapse‐free survival (RFS) were calculated using the Kaplan–Meier method and survival curves were compared using the log‐rank test. Survival was calculated from 2 years after transplantation until death or last follow‐up. Expected survival for a comparable general population was estimated using the Ederer2‐estimator in combination with mortality rates from a general lifetable stratified by sex, age (1‐year groups) and calendar year (1‐year groups) [10]. The incidence of transplant‐related mortality (TRM) and relapse were estimated using the competing risk analysis by Fine and Gray‐considering relapse as a competing event for TRM and death without relapse as competing event for relapse. Patients were censored at the time of last follow‐up, 31 Jan 2021.

Uni‐ and multivariate predictive analyses for relapse and TRM were performed with the proportional sub‐distribution hazard regression model of Fine and Gray, while analysis of OS and RFS were performed using the Cox proportional hazards model.

Factors analysed: patient and donor age, gender and pre‐HSCT cytomegalovirus (CMV) sero‐status, diagnosis, gender match, HLA‐match, donor type, stem cell source, CD34+ cell dose, conditioning intensity, ATG, GVHD prophylaxis, previous acute and chronic GVHD, previous blood‐stream infection, invasive fungal infection and CMV reactivation.

Since acute GVHD, IFI and BSI antedated the 2 years analysis, these factors were treated as non‐time‐dependent factors. Most (95%) chronic GVHD already existed at 2 years, and only a few patients (*n* = 15, 8 limited and 7 extensive) developed chronic GVHD beyond 2 years. For this reason, we treated chronic GVHD as a time‐dependent covariate in the Cox proportional hazards model. The proportional hazards assumptions were tested with scaled Schoenfeld residuals. Analyses were performed using the EZR freely available software, and Statistica 13 (Tulsa, OK, USA) and Stata version 16.1 software.

## 3 RESULTS

Of 1,000 adult patients with a malignant disease transplanted during the study period, 421 were alive and disease free 2 years post‐transplant. Median follow‐up were 6.2 years (2.0–16.1).

By the end of follow‐up, 69 (16%) patients had died.

In this cohort, 40 patients (10%) had suffered previous acute GVHD grades III–IV, 137 (33%) extensive chronic GVHD, 253 (60%) BSI, 163 (39%) CMV reactivation, and 50 (12%) probable or proven IFI prior to start of the study period.

### 3.1 Survival

**FIGURE 1:**
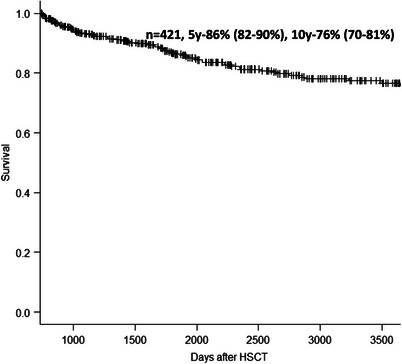
Overall survival in patients alive without relapse 2 years after HSCT. HSCT, haematopoietic stem cell transplantation.

Five and 10 years survival from HSCT were 86% (95% CI: 82%–90%) and 76% (70%–81%), respectively (Figure 1). Causes of death were: relapse 22 (5.2%), GVHD 19 (4.5%), infection 8 (1.9%), secondary malignancy 9 (2.1%), suicide 2 (0.5%), multi‐organ failure 2 (0.5%), and various other causes 7 (1.7%). Factors associated with increased mortality in multivariate analysis were previous BSI (HR; 2.48, 95% CI; 1.27–4.88, *p* < 0.01), IFI (2.85, 1.65–4.94, *p* < 0.001), extensive chronic GVHD (time‐dependent) (3.79, 2.21–6.51, *p* < 0.001), CLL (5.09, 1.93–13.4, *p* = 0.001), CMV sero‐negative donor (2.26, 1.32–3.85, *p* = 0.003), and patient age ≥ 60 years (1.78, 1.03–3.07, *p* = 0.038) (Table 2).

**TABLE 2:** Results from multivariate analyses (MVA) of factors affecting outcome in patients alive without relapse 2 years after HSCT.

Factor	MVA OS	MVA NRM	MVA RI
**Age ≥ 60 years vs. < 60 years**	1.78, 1.03–3.07, 0.038	3.14, 1.67–5.90, < 0.001	–
**Donor CMV sero − vs. +**	2.26, 1.32–3.85, 0.003	–	4.39, 1.67–11.5, 0.003
**BSI vs**. no BSI	2.48, 1.27–4.88, 0.008	2.22, 1.00–5.01, 0.049	–
**IFI vs**. no IFI	2.85, 1.65–4.94, < 0.001	2.55, 1.31–4.95, 0.006	–
**Ext cGVHD vs**. no + limited^*^	3.79, 2.21–6.51, < 0.001	6.38, 3.01–13.5, < 0.001	–
**Diagnose**:			
ALL	1.45, 0.60–3.52, 0.41	1.61, 0.46–5.64, 0.46	1.33, 0.45–3.95, 0.60
CML	0.23, 0.03–1.72, 0.15	0.48, 0.06–3.62, 0.47	0.00, 0.00–0.00, 0.99
Lymphoma	1.78, 0.98–3.23, 0.06	3.55, 1.76–7.17, < 0.001	0.36, 0.08–1.55, 0.17
CLL	5.09, 1.93–13.4, 0.001	9.22, 3.24–26.2, < 0.001	5.07, 1.48–17.3, 0.01
MDS/MPN	0.89, 0.40–1.95, 0.76	1.24, 0.48–3.19, 0.66	0.57, 0.17–1.93, 0.37
AML	Ref.	Ref.	Ref.

*Note*: Hazard ratio (HR), 95% confidence interval (CI) and *p*‐values are displayed for the various variables.

Abbreviations: ALL, acute lymphoblastic leukaemia; AML, acute myeloid leukaemia; BSI, blood‐stream infection; CLL, chronic lymphocytic leukaemia; CML, chronic myeloid leukaemia; Ext cGVHD, extensive chronic graft‐versus‐host disease; IFI, invasive fungal infection; MDS/MPN, myelodysplastic syndrome/myeloproliferative neoplasm; MVA, multivariate analysis; NRM, non‐relapse mortality; OS, overall survival; RI, relapse incidence.

^*^ Analysed as a time‐dependent variable.

**FIGURE 2:**
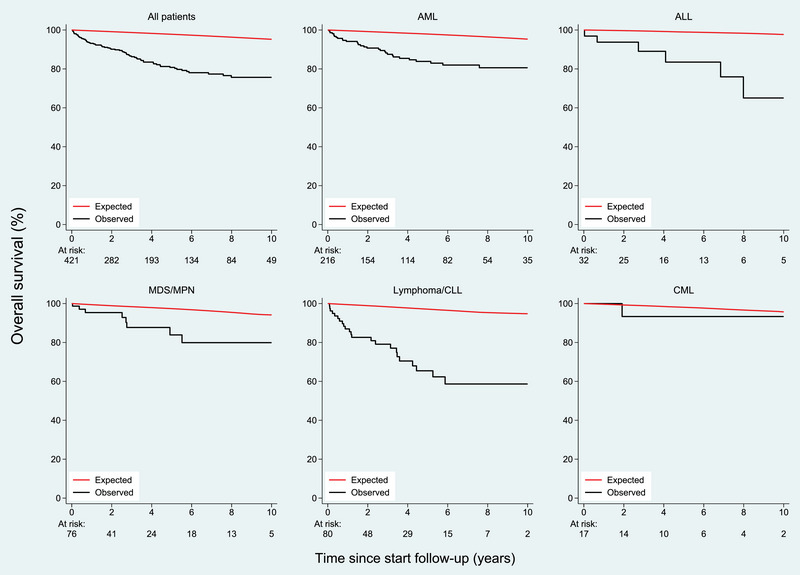
Overall survival in patients alive without relapse 2 years after HSCT compared with Norwegian age‐ and gender‐matched population. HSCT, haematopoietic stem cell transplantation.

Other factors significant only in univariate analysis were: conditioning intensity, stem cell source and acute GVHD. The 5 years OS for patients with chronic myeloid leukaemia (CML) were 93%, acute myeloid leukaemia (AML) 88%, myelodysplastic syndrome/myeloproliferative neoplasm (MDS/MPN) 88%, acute lymphoblastic leukaemia (ALL) 89%, lymphoma 83% and chronic lymphocytic leukaemia (CLL) 57% (*p* = 0.006) (Figure 2). The 10 years OS for patients receiving RIC and MAC were 62% and 81%, *p* = 0.001.

### 3.2 Non‐relapse mortality

**FIGURE 3:**
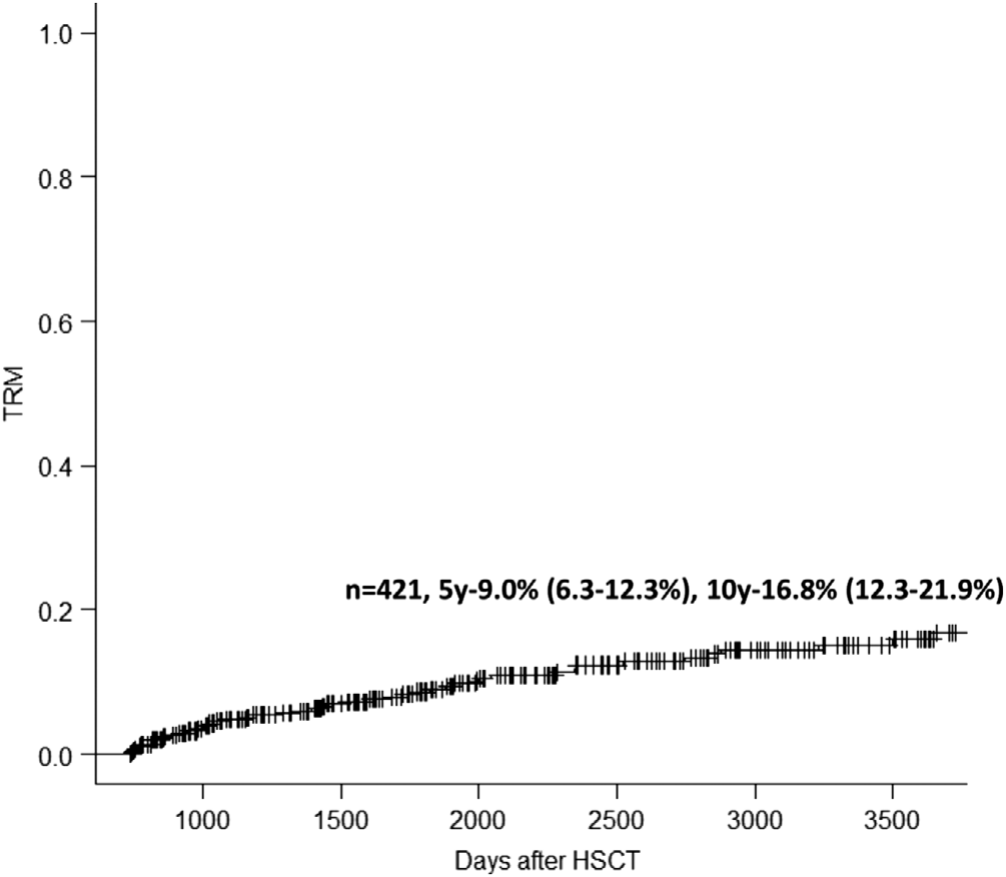
Transplant‐related mortality (TRM) in patients alive without relapse 2 years after HSCT. HSCT, haematopoietic stem cell transplantation.

For patients alive without relapse 2 years after HSCT the 5 and 10 years NRM (from HSCT) were 9.0% (6.3%–12.3%) and 16.8% (12.3%–21.9%) (Figure 3).

Factors associated with increased NRM were age ≥ 60 years (3.14, 1.67–5.90, *p* < 0.001), a previous BSI (2.22, 1.00–5.01, *p* < = 0.05), previous IFI (2.55, 1.31–4.95, *p* = 0.006), extensive chronic GVHD (6.38, 3.01–13.5, *p* < 0.001), lymphoma (3.55, 1.76–7.17, *p* < 0.001) and CLL (9.22, 3.24–26.2, *p* < 0.001) (Table 2).

The 5 years NRM for patients with CML were 6.7%, AML 6.6%, MDS/MPN 8.2%, ALL 3.1%, lymphoma 15.3% and CLL 42.9%, *p* < 0.001. In patients treated with ATG (thymoglobulin) (*n* = 81) no NRM occurred.

### 3.3 Relapse

**FIGURE 4:**
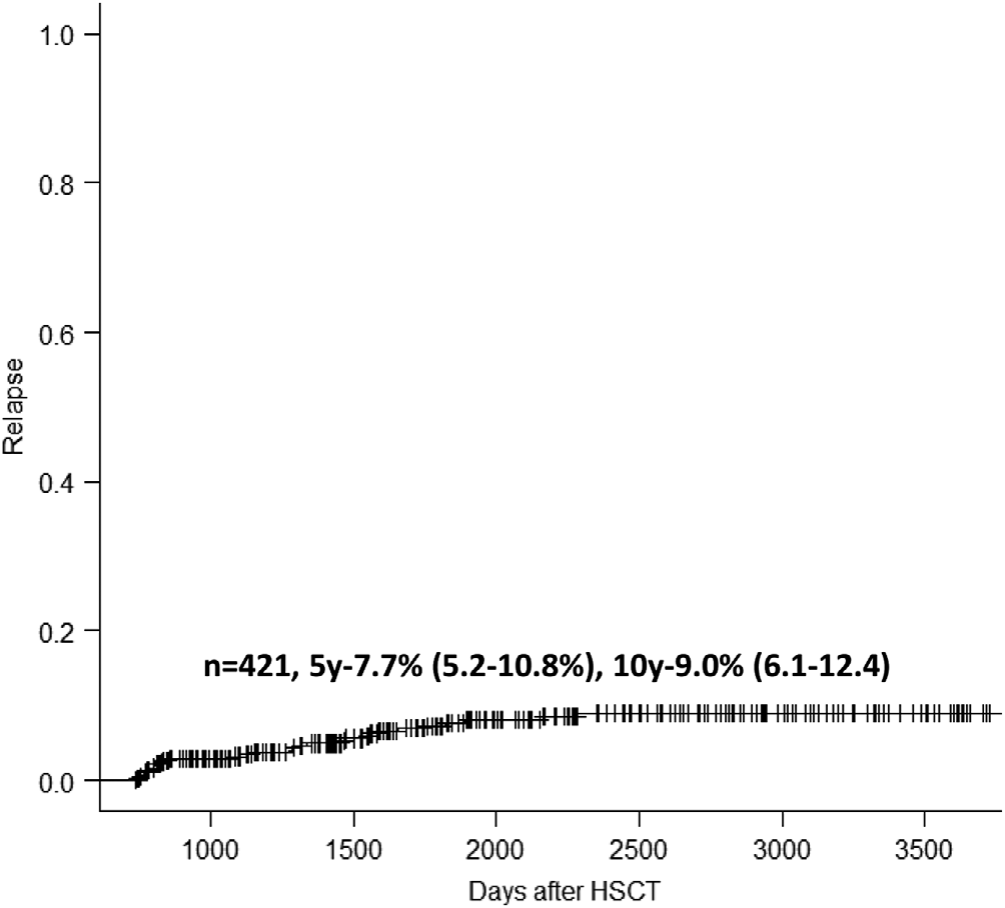
Relapse incidence in patients alive without relapse 2 years after HSCT. HSCT, haematopoietic stem cell transplantation.

For patients alive without relapse 2 years after HSCT, the 5 and 10 years relapse incidence (from HSCT) were 7.7% (5.2%–10.8%) and 9.0% (6.1%–12.4%) (Figure 4).

Two factors were associated with increased relapse incidence: a CMV sero‐negative graft (4.39, 1.67–11.5, *p* = 0.003) and CLL (5.07, 1.48–17.3, *p* = 0.01) (Table 2).

The 5 years relapse incidence for patients with CML were 0%, AML 9.1%, MDS/MPN 3.7%, ALL 13.8%, lymphoma 3.1% and CLL 19.0%.

Most relapses (27/30) occurred between 2 and 5 years after HSCT, while only three occurred beyond 5 years post HSCT.

The proportional hazard assumption was not fulfilled in the analysis of CLL diagnosis due to crossing K–M curves. For this reason, we split the follow‐up period into 2‐year intervals. However, the number of patients with CLL was very limited (*n* = 12) and events (relapse) were few (*n* = 3) making the interpretation very doubtful.

## 4 DISCUSSION

In this study, we analysed outcome of patients alive without relapse 2 years after HSCT. Acceptable long‐term survival was achieved with 86% and 76% at 5 and 10 years, respectively. Results varied across different diagnosis. Only patients transplanted for CML were in accordance with survival in the Norwegian age‐ and gender‐matched population. Superior long‐term outcome for CML after HSCT has been reported previously [11]. Dismal prospects for lymphoproliferative disorders after HSCT, CLL in particular, are in our material as well as others [12] mainly due to high NRM; 36% among our patients with chronic GVHD being the main culprit. Compared with two larger previous studies of patients alive and free of disease at 2 years post HSCT, one from 1999 [13] with probability of surviving 5 years at 89% and the other from 2011 with 85% at 10 years [9], our results are slightly inferior. However, cited studies included only transplants with MAC, recipients were young with a median age of 16–34 years, and non‐malignant diseases as aplastic anaemia were part of the cohort. Our population had a higher median age of 50 years, nearly half of them received RIC with PBSC as stem cell source, only malignant diagnoses were included, and in contrast to the 1999 study with CML making up nearly one‐third of the patients only 4% were patients with CML in our cohort. These are factors known to be associated with increased risk of relapse or chronic GVHD, which is a likely explanation for the difference in OS. A more recent single centre analysis from 2018 with patient characteristics comparable to ours reported 5 years OS at 78% in a cohort of patients alive and free of relapse at 1 year after HSCT [14].

Factors associated with impaired late OS and NRM were mainly transplant related complications occurring during the two first years after HSCT: chronic GVHD, BSI and IFI. Age was of significance with inferior prospects for older patients. These are factors known to increase early mortality [15, 16], and chronic GVHD and age being major contributors to late mortality are well established [14, 17]. Regarding BSI, several studies have addressed this issue: the largest from 2019 showing an association between BSI by day 100 and impaired 1‐year survival [18]. Here, we show that BSI also has negative impact on long‐term survival. Reasons may be numerous. BSI may be a surrogate marker for comorbidity and frailty. Further, early BSI is associated with acute GVHD [19] either manifest or subclinical, facilitating thymus injury either from allo‐reactive T‐cells and/or immunosuppressant drugs, corticosteroids in particular [20]. Net result being impaired function for thymus epithelial cells with reduced thymopoiesis and negative selection, resulting in impaired regeneration of T‐cells and reduced elimination of allo‐reactive T‐cells, negatively affecting adaptive immunity [21]. This sets the scene for long‐term complications such as immunodeficiency, reduced GVL with subsequent relapse, or release of allo‐reactive T‐cells resulting in chronic GVHD [22]. Another possible mechanism may be through loss of gut microbiota diversity after BSI treatment with broad‐spectrum antibiotics. High microbiota diversity during engraftment is associated with superior survival compared to low diversity, with positive impact on NRM and less GVHD in the former group [23, 24]. Interestingly, recent data indicate that gut microbiota influence immune reconstitution, and that low microbiota diversity may impair neutrophil and lymphocyte recovery [25, 26].

Regarding IFI, increased NRM for up to 2 years has been reported, and risk factors for IFI such as impaired immune reconstitution, acute GVHD, and subsequent prolonged immunosuppressive therapy may lead to impaired GVL with relapse or chronic GVHD in the long run [27, 28]. Thus, IFI may serve as a surrogate marker for factors associated with risk for long‐term mortality. As for BSI, here we show that IFI is associated with increased long‐term mortality. Taken together, early BSI and IFI are associated with increased long‐term NRM where measures for optimising prophylaxis, treatment, immune reconstitution, and microbiota may be warranted, and should be addressed in future studies.

Relapse was the leading cause of late deaths (32% of all deaths) in line with comparable studies [9, 29]. Nearly all relapses (27/30) occurred between years 2 to 5 after HSCT, and relapse beyond that time point was very rare. In multivariate analysis, only CLL and CMV negative donor were of statistical significance. CMV latency or reactivation and its association with enhanced GVL effect and reduction in short‐term relapse incidence has been shown in several publications with the proposed main mechanism being CMVs induction of NKG2C+/NKG2A− NK cells with NKG2C mediated cytotoxicity against HLA‐E expressing malignant cells [30, 31, 32]. There is evidence of a similar response by a subset of γδ T‐cells to an undefined antigen, but the role of αβ T‐cells in this setting is less clear [33]. Our findings indicate that this antitumour activity also holds long‐term. In our cohort, the protective effect of a CMV‐positive donor was present regardless of manifest CMV‐reactivation or not, and regardless of recipient serostatus (data not shown). Choosing a CMV+ donor for CMV+ recipient is supported, but in the era of improved CMV monitoring, CMV treatment, and reduction in risk factors for CMV‐reactivation such as better GVHD‐prophylaxis with ATG or PTCy, we hypothesise that the protective effect of a CMV‐positive donor might also warrant a CMV+ donor for a CMV− recipient. However, this should be addressed in future studies.

Other major causes of death were chronic GVHD (28%), secondary malignancies (13%) and infections (12%). The importance of the ever prominent chronic GVHD is of no surprise and well documented [8, 9]. Thymus injury from radio‐ and/or chemotherapy as stated above [21] is a risk factor. This might explain the high incidence among HSCT CLL and lymphoma patients in our cohort, being a particularly heavily pre‐treated group, many transplanted in third remission or even beyond. The incidence of GVHD is fortunately decreasing, in particular related to increased use of ATG or post‐transplantation cyclophosphamide (PTCy) as is our experience [34] as well as that of others [35, 36]. Increased risk of death due to secondary malignancies for long‐term survivors after HSCT, another known significant complication [37] may be even more apparent as HSCT survivors live longer. However, changes in treatment protocols with less toxic conditioning regimens, for example, less use of TBI, measures for optimising immune reconstitution, reduction in chronic GVHD with subsequent less use of immunosuppressive drugs, and adequate guidelines for screening and long‐term follow‐up may reduce the incidence of secondary malignancies.

Our findings must be interpreted with caution. This is a single centre study with limited number of patients, and transplantation practices have changed over the 14 years spanning the study period. In particular, use of ATG and PTCy as GVHD prophylaxis, implementation of haploidentical donors and steadily increasing number of older patients with use of RIC protocols may affect future outcome.

## 5 CONCLUSION

The prospect for long‐term survival is good for 2‐year survivors of HSCT. However, life expectancy remains inferior to the general population. Optimising prophylaxis and treatment for chronic GVHD, BSI and IFI are needed, along with guidelines for follow‐up regarding secondary malignancies. Measures to improve immune reconstitution, microbiota, and use of CMV seropositive donors regardless of donor serostatus may be warranted.

## AUTHOR CONTRIBUTIONS

**M. Remberger, T. Gedde‐Dahl, B. Grønvold**: Conception and design. **All authors**: Provision of study materials or patients; collection and assembly of data; data analysis and interpretation; manuscript writing; final approval of manuscript.

## CONFLICT OF INTEREST STATEMENT

The authors declare no conflicts of interest.

## ETHICS STATEMENT

The study was approved by the Regional Committee for Medical and Health Research Ethics of South‐East Norway. The procedures were in accordance with the Helsinki Declaration.

## PATIENT CONSENT STATEMENT

Consent form including permission for publication is signed by all patients included.

## PERMISSION TO REPRODUCE MATERIAL FROM OTHER SOURCES

No copyrighted work from previously published materials is included in this paper.

## Data Availability

The data supporting the findings of this study are not openly available due to reasons of sensitivity and are available from the corresponding author upon request. Data are located at Oslo University Hospital in controlled access.
